# Hand Stereotypies in Rett Syndrome

**DOI:** 10.15844/pedneurbriefs-34-2

**Published:** 2020-02-12

**Authors:** Matheus G. Ferreira, Hélio A. G. Teive

**Affiliations:** 1 Movement Disorders Unit, Neurology Service, Internal Medicine Department, Hospital de Clínicas, Federal University of Paraná, Curitiba, PR, Brazil

**Keywords:** Motor Stereotypies, Rett Syndrome, Movement Disorders

## Abstract

Researchers from the Rett Syndrome Natural History Study (RNHS) present longitudinal data across the United States of America aimed to characterize hand stereotypies (HS) in this large cohort of patients with Rett syndrome.

Researchers from the Rett Syndrome Natural History Study (RNHS) present longitudinal data across the United States of America aimed to characterize hand stereotypies (HS) in this large cohort of patients with Rett syndrome. They reported 922 patients with classic Rett syndrome, 75 with atypical severe and 77 with atypical mild Rett syndrome. All patients were female and were assessed every 6 to 12 months between 2006 to 2015. The comparison group consisted of 49 patients who did not meet the clinical criteria for Rett syndrome but had documented MECP2 mutations. MECP2 mutations were classified according to severity in mild, moderate or severe. Hand stereotypies were pre-classified in 8 groups. The authors detected HS in 99.5% of Rett syndrome patients at enrollment against only 35% among the non-Rett group, confirming the specificity of thus clinical finding.

The authors detected HS in 99.5% of Rett syndrome patients at enrollment against only 35% among the non-Rett group, confirming the specificity of this clinical finding. Hand mouthing and clapping/tapping were more frequently found than wringing/washing. No difference was observed when comparing hand stereotypies prevalence and specific mutations in MECP2; there was, however, an association between severity of MECP2 mutation and a higher frequency and number of stereotypies. Prevalence and frequency of hand stereotypies did not differ when comparing patients younger than 21 years to participants 21 years and older. The number stereotypies and severity of mouthing was higher among the pediatric population. Age of onset was remarkably different between study categories, with atypical severe patients having an earlier onset (1.52 ± 1.1 years) compared to typical Rett syndrome (1.87 ± 1.1 years) and atypical mild patients (3.06±2.5years; p<0.001). Additionally, the majority of Rett syndrome patients showed developmental regression first and later developed hand stereotypies (62.7%).

Finally, the presence of hand stereotypies was not related to disease severity or other characteristics. The longitudinal analysis of this study showed that while the level of hand function seems to relate to the age at onset and frequency of hand stereotypies, the progressive decline in manual abilities does not follow the same path. In fact, the loss of function should be analyzed in a broader context considering other features of the disease, such as rigidity and bradykinesia. [1]

COMMENTARY. Motor stereotypies are common childhood onset movement disorders with complex aetiologies [2]. By the beginning of this decade Edwards et al proposed it to be defined as “*a non-goal-directed movement pattern that is repeated continuously for a period of time in the same form and on multiple occasions, and which is typically distractable*” [3]. Hand stereotypies are a defining characteristic of Rett syndrome, confirmed by this paper. The present study however has two very interesting aspects, especially for a rare disease: its longitudinal design and a large number of patients. Here the authors explore from different angles hand stereotypies, probably the most remarkable clinical sign of the disease. The identification of the multiple causative MECP2 mutations [4] has aided in understanding the phenotypical variety observed in clinical practice. Interestingly, they found that severe mutations correlated with a greater number of stereotypies and not with their severity. These findings may help identify a genotype-phenotype correlation related to each specific mutation, however more data is necessary.

It should be observed, that in this article, adults represented only 12% of the population studied and male patients were excluded. Although these are infrequent clinical findings, it is reasonable to expect such cases as clinical care and diagnostic tools become more available throughout the world. Hence, a focused analysis including these groups is necessary in future studies.

## Disclosures

The authors have declared that no competing interests exist.
